# Targeting Siderophore Biosynthesis to Thwart Microbial Growth

**DOI:** 10.3390/ijms26083611

**Published:** 2025-04-11

**Authors:** Beatriz M. Rocha, Eugénia Pinto, Emília Sousa, Diana I. S. P. Resende

**Affiliations:** 1 LQOF—Laboratório de Química Orgânica e Farmacêutica, Departamento de Ciências Químicas, Faculdade de Farmácia, Universidade do Porto, Rua de Jorge de Viterbo Ferreira 228, 4050-313 Porto, Portugal; 2 CIIMAR/CIMAR LA—Centro Interdisciplinar de Investigação Marinha e Ambiental, Universidade do Porto, Terminal de Cruzeiros do Porto de Leixões, 4450-208 Matosinhos, Portugal; 3 Laboratório de Microbiologia, Departamento de Ciências Biológicas, Faculdade de Farmácia, Universidade do Porto, Rua de Jorge de Viterbo Ferreira 228, 4050-313 Porto, Portugal; 4 ICBAS—Instituto de Ciências Biomédicas Abel Salazar, Universidade do Porto, Rua de Jorge de Viterbo Ferreira 228, 4050-313 Porto, Portugal

**Keywords:** siderophores, iron acquisition, antimicrobial resistance, biosynthesis inhibition, drug discovery

## Abstract

The growing threat of antibiotic resistance has made treating bacterial and fungal infections increasingly difficult. With the discovery of new antibiotics slowing down, alternative strategies are urgently needed. Siderophores, small iron-chelating molecules produced by microorganisms, play a crucial role in iron acquisition and serve as virulence factors in many pathogens. Because iron is essential for microbial survival, targeting siderophore biosynthesis and transport presents a promising approach to combating drug-resistant infections. This review explores the key genetic and biochemical mechanisms involved in siderophore production, emphasizing potential drug targets within these pathways. Three major biosynthetic routes are examined: nonribosomal peptide synthetase (NRPS)-dependent, polyketide synthase (PKS)-based, and NRPS-independent (NIS) pathways. Additionally, microbial iron uptake mechanisms and membrane-associated transport systems are discussed, providing insights into their role in sustaining pathogenic growth. Recent advances in inhibitor development have shown that blocking critical enzymes in siderophore biosynthesis can effectively impair microbial growth. By disrupting these pathways, new antimicrobial strategies can be developed, offering alternatives to traditional antibiotics and potentially reducing the risk of resistance. A deeper understanding of siderophore biosynthesis and its regulation not only reveals fundamental microbial processes but also provides a foundation for designing targeted therapeutics. Leveraging these insights could lead to novel drugs that overcome antibiotic resistance, offering new hope in the fight against persistent infections.

## 1. Introduction

**Figure 1:**
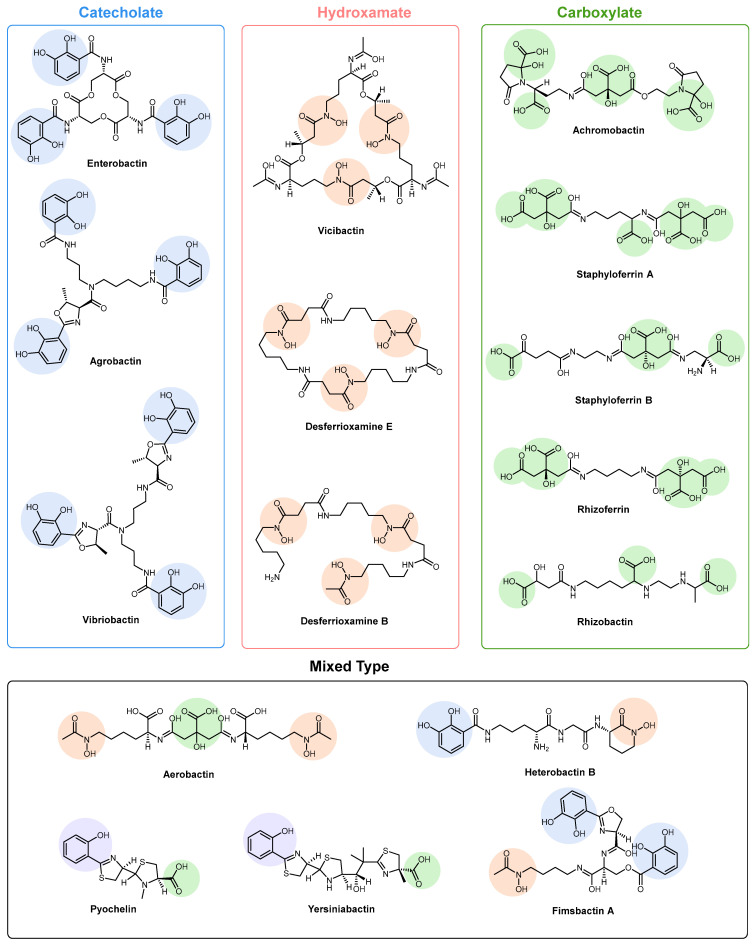
Representative classes of siderophores (catecholate groups are highlighted in blue, hydroxamate in pink, carboxylate in green, and phenolates in purple).

Microbial resistance is currently one of the most relevant public health problems globally, as it presents worrying clinical and economic consequences and is often associated with the inappropriate use of antimicrobials [1]. A noticeable correlation exists between increased antibiotic consumption and elevated levels of microbial resistance [2,3]. Given the significant impact of microbial resistance on global public health, organizations such as the European Commission and the World Health Organization have acknowledged the importance of studying the emergence of microbial resistance. They advocate for the development and implementation of surveillance and control systems, such as the European Microbial Resistance Surveillance System [4,5,6]. As a result, research efforts have shifted towards exploring alternative strategies to traditional antibiotics, focusing not only on identifying new targets and mechanisms of action but also on developing innovative methods to overcome resistance [7,8]. One of these strategies is through the manipulation of the microbial iron transport pathways to deliver antimicrobials to the cell using siderophores [8]. Siderophores are metabolites produced and secreted by various organisms, including fungi, bacteria, and plants [9,10]. These metabolites are capable of scavenging iron from the extracellular environment to make it available intracellularly [11,12]. Siderophores bind to insoluble Fe(III) and are subsequently conveyed into microbial cells via membrane-bound iron siderophore receptors [10]. Following this, the iron is typically released from the siderophores through the microbial-mediated reduction of Fe(III) to Fe(II) via redox processes. This iron is essential for various biological processes in microorganisms as it plays a crucial role in deoxyribonucleic acid (DNA) synthesis, as iron-dependent enzymes like ribonucleotide reductase require it to produce deoxyribonucleotides [13,14]. Iron is also a key component of electron transport chains, supporting cellular respiration and adenosine triphosphate (ATP) production [15]. Additionally, it is involved in enzyme function, acting as a cofactor in numerous metabolic pathways, including oxidative stress defence and nitrogen fixation [16]. Iron acquisition by siderophores represents a key survival mechanism in iron-limited environments, with iron homeostasis being essential for cellular growth and immune responses [17,18]. Three categories of siderophores can be differentiated according to the components involved in iron chelation [9,12]. They include catecholates (i.e., enterobactin, agrobactin, and vibriobactin, Figure 1), hydroxamates (i.e., vicibactin, desferrioxamine E, and desferrioxamine B, Figure 1), and carboxylates, including α-hydroxycarboxylates (i.e., achromobactin, staphyloferrin A, staphyloferrin B, rhizoferrin, and rhizobactin, Figure 1) [12,19,20,21]. Mixed-type siderophores (i.e., aerobactin, heterobactin B, pyochelin, yersiniabactin, fimsbactin A, Figure 1) exhibit structural features corresponding to two or three classes simultaneously and may include a fourth siderophore type, phenolates [20]. All siderophores belonging to the different categories possess distinct properties influencing their affinity for iron [22]. The strong affinity and selectivity of siderophores for Fe(III) result from the meticulous choice of metal-binding groups, the abundance of binding units, and their stereochemical arrangement. Oxygen atoms are the primary donors for Fe(III) coordination, with the most common coordination geometry being octahedral, where six heteroatoms surround the iron centre. The preference toward octahedral coordination facilitates the creation of thermodynamically stable high-spin Fe(III) species.

Within catecholate-type siderophores, the Fe(III) ion forms bonds with phenol or catecholate groups. This bonding leads to the creation of a hexadentate–octahedral complex, where two oxygen atoms from each catecholate group are engaged [9]. Hydroxamate siderophores are characterized by the structure C(=O)N-(OH)R, where R represents an amino acid or its derivative. This R group contains two oxygen atoms, forming a bidentate ligand with Fe ions. While every siderophore exhibits a greater affinity for Fe(III) compared to Fe(II), desferrioxamine B (Figure 1), a hexadentate siderophore containing three hydroxamate units, demonstrates an Fe(III) affinity that exceeds its affinity for Fe(II) by 20 orders of magnitude. The relatively low affinity for Fe(II) allows for an efficient release mechanism inside the cell, typically through reduction processes [23]. When hydroxamate binds with Fe(III), its functional group undergoes deprotonation, specifically losing a proton from the hydroxylamine group (-NOH), resulting in the formation of a bidentate ligand [20,24].

While the classification and characterization of the primary types of siderophores are crucial for identifying how different microorganisms utilise siderophores to scavenge iron from their environment [9,19], a process vital for their survival and pathogenicity, understanding the biosynthetic pathways [24,25,26,27] and iron acquisition mechanisms [23,25,28,29] sheds light on the fundamental biological processes and reveals potential vulnerabilities that can be exploited for therapeutic interventions [30]. Comprehending the biosynthetic pathways and iron acquisition mechanisms unveils the complex processes through which siderophores are synthesized, secreted, and subsequently utilized by microorganisms. Knowing these pathways not only enlightens us of the fundamental biological processes, like the role of specific transporters and receptors in facilitating iron acquisition, which is essential for various cellular functions but also reveals potential vulnerabilities that can be exploited for therapeutic interventions.

The structural features, general biology [19,31], and methods for the detection, isolation, quantification, and characterization [32] of siderophores have been extensively reviewed in the literature. Detailed reviews have also been published on the siderophore’s biosynthesis [25,26,27], including specific works focused on the iron acquisition mechanisms [28]. Nevertheless, the inhibition of specific enzymes responsible for the biosynthesis of siderophores has scarcely been explored, with few original research works focusing on individual enzymes. The existing literature on this topic has not been compiled or analysed in a single document. Hence, this review aims to update the existing knowledge on the potential therapeutic targets within siderophore biosynthesis pathways. It emphasizes the importance of inhibiting enzymatic catalytic sites as a promising strategy to combat antimicrobial resistance, offering new avenues for the development of novel antimicrobials.

## 2. Siderophore Biosynthesis

Siderophore biosynthesis is segmented into three main pathways: nonribosomal peptide synthetase (NRPS)-dependent, polyketide synthase (PKS), and NRPS-independent siderophore (NIS) synthetase [33]. Siderophores are commonly produced by NRPS or PKS domains, often in conjunction with NRPS modules. Additionally, a minority of siderophores are synthesized via pathways that operate independently of NRPS and PKS [24,25,34].

### 2.1. Nonribosomal Peptide Synthetase (NRPS) Biosynthesis

**Figure 2:**
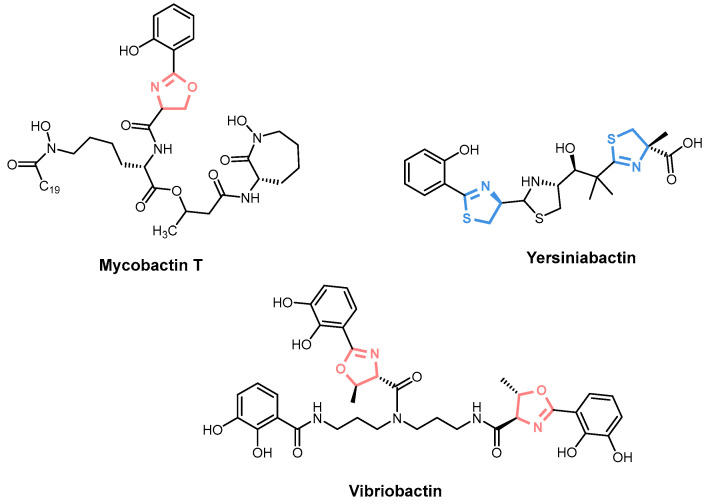
Examples of NRPS siderophores: mycobactin T, yersiniabactin, and vibriobactin with oxazoline rings highlighted in pink and thiazoline rings shown in blue.

The predominant method of siderophore synthesis involves NRPS, which orchestrates a multienzyme assembly process. Examples of NRPS siderophores include mycobactin T, yersiniabactin, and vibriobactin (Figure 2), each distinguished by characteristic oxazoline or thiazoline rings [34].

**Figure 3:**
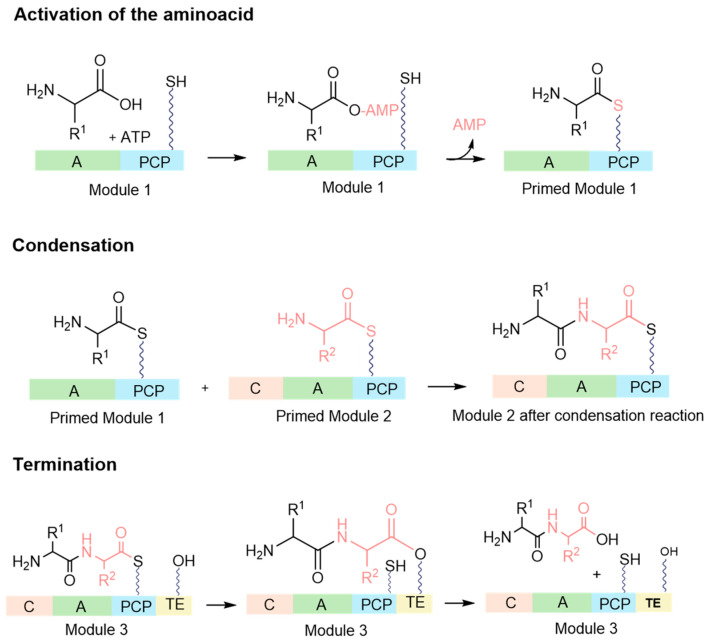
Siderophore biosynthesis by NRPS. Green: adenylation domain (A); blue: peptidyl carrier protein (PCP); orange: condensation domain (C); yellow: thioesterase domain (TE) (adapted from [24,34]).

NRPSs are extensive, multimodular enzyme complexes comprising adenylation (A), thiolation (T), condensation (C), and thioesterase (TE) domains, as well as other specific functional domains, including epimerization (E), oxidation (Ox), methylation (Mt), and cyclization (Cy) [35,36]. During this process, amino acids, along with carboxy and hydroxy acids, are assembled into a peptidic precursor molecule [37]. The process begins with the activation of the amino acid (Figure 3), converting it into an amino acyl-adenosine monophosphate (AMP) through the adenylation domain [38]. Subsequently, the activated amino acid is covalently linked (releasing the AMP in the process) to the carrier domain, thus preparing the module. The carrier domain’s ability to accept the amino acid stems from a prior post-translational modification, where a phosphopantetheinyl moiety (a prosthetic group of several acyl carrier proteins) is added onto a conserved serine residue [19]. Once two neighbouring modules are primed, the condensation domain (Figure 3) facilitates the formation of a peptide bond between the two amino acids, resulting in a dipeptide on the second carrier domain. With the priming of the third carrier domain, a subsequent peptide bond is created, yielding a tripeptide on the third carrier domain. This sequential assembly process persists until the chain reaches completion [34]. The final stage involves either assembling or releasing chains from the NRPS through hydrolysis or cyclization. The cleavage of the acyl thioester, which is bound to the T domain, is facilitated by a 1,4-dihydronicotinamide adenine dinucleotide (NADH)-dependent reaction (Figure 3) [22,39].

### 2.2. Polyketide Synthase (PKS) Biosynthesis

**Figure 4:**
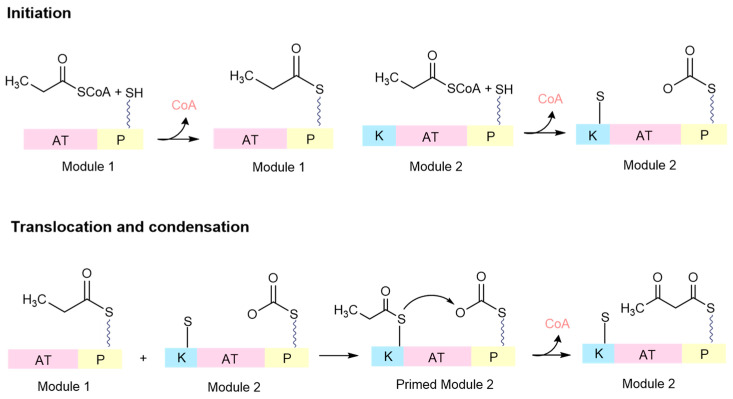
Siderophore biosynthesis by polyketide synthase. Pink: acyltransferase domain (AT); yellow: acyl carrier protein (P); blue: ketosynthase domain (K).

Certain siderophores are produced through polyketide synthases. The PKS module comprises a ketosynthase domain (K), an acyltransferase domain (AT), and a carrier domain (P). The acyltransferase domain covalently binds the initiation unit to the carrier domain, releasing CoA (Figure 4). Likewise, in module 1, the elongation unit is attached similarly. In both instances, the modules are primed to accept the chain through a specific biochemical process known as phosphopantetheinylation—a kind of molecular ‘switch’ that equips carrier proteins for their role in the NRPS system. This activation allows for the transfer of the acyl chain from the carrier domain of the loading module to a cysteine residue in the ketosynthase domain, effectively priming module 1 for chain elongation (Figure 4) [19]. Subsequently, the ketosynthase domain catalyses the condensation reaction, attaching the growing chain to the carrier domain of the first module, ready for translocation to the ketosynthase domain of the next module. This assembly line process persists, with various tailoring domains integrated into each module to facilitate the addition of diverse functionalities. Finally, a thioesterase domain, through reduction, hydrolysis, or sometimes cyclization, releases the complete chain from the final carrier domain [19,22,34].

### 2.3. NRPS-Independent Siderophore (NIS) Synthetase Biosynthesis

**Figure 5:**
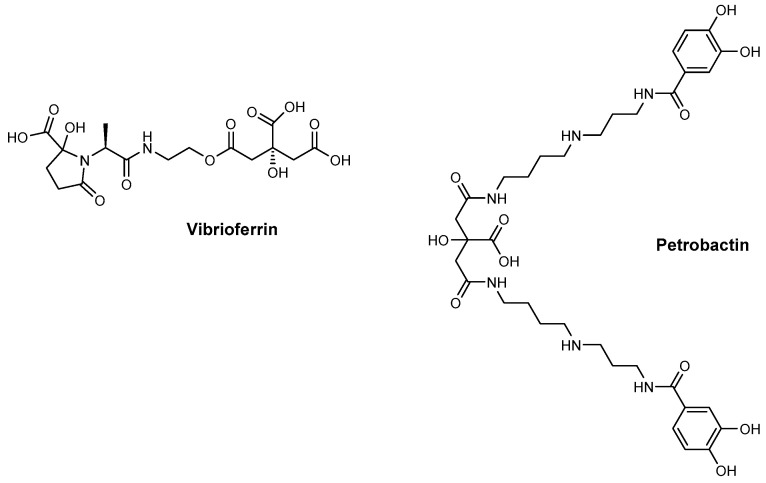
Structures of vibrioferrin and petrobactin.

NISs play a crucial role in the creation of various linear and cyclic siderophores, such as aerobactin (Figure 1), desferrioxamine (Figure 1), petrobactin (Figure 5), and vibrioferrin (Figure 5) [34]. Hydroxamate and carboxylate siderophores are primarily assembled through NRPS-independent pathways. The synthesis of these siderophores typically involves a variety of enzymes, including monooxygenases, decarboxylases, aminotransferases, ac(et)yltransferases, amino acid ligases, and aldolases [40]. These enzymes facilitate the conjugation of a carboxylate and a nucleophile, typically an amine or occasionally an alcohol, thereby creating a peptide or ester bond. NIS synthetases produce siderophores incorporating citric acid, α-ketoglutarate, or succinic acid [34]. Within the NIS synthetase, an acyladenylation domain is responsible for generating compounds like citrate–AMP, which supplies high-energy bonds essential for the subsequent condensation reaction with amino acids or polyamines [24].

**Figure 6:**
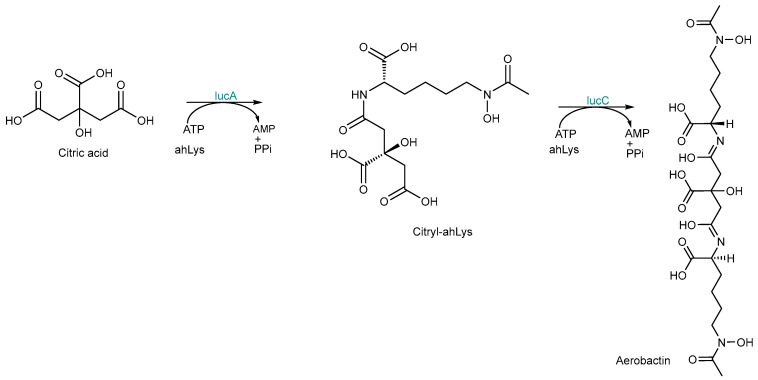
Aerobactin biosynthetic pathway. IucA generates 3*S*-citryl-ahLys, which serves as the substrate for IucC to produce aerobactin with an achiral citryl backbone. ahLys-*N*^6^-acetyl-*N*^6^-hydroxy-L-lysine.

As previously mentioned, aerobactin is synthesized through the NIS pathway. IucA and IucC are essential components of the operon involved in aerobactin biosynthesis, initially discovered and characterized in *Escherichia coli*. These enzymes are part of the ligase family known as NIS synthetases [41,42]. IucA is the archetypal Type A NIS synthetase, utilizing citrate as its substrate, while IucC is classified as a Type C NIS synthetase, which acts on a derivative of citrate [42]. The biosynthetic pathway for aerobactin is illustrated in Figure 6. Initially, the carboxylate undergoes activation through ATP reaction, forming an acyladenylate. Subsequently, the amine substrate displaces AMP to yield the product. Noteworthily, these reactions take place outside ribosomes and in the absence of large protein complexes. Instead, NIS siderophores are synthesized within small, soluble proteins devoid of homology to any other enzymes. However, the biosynthesis of many NIS siderophores requires the activity of more than one NIS enzyme. An instance of a siderophore created through the collaboration of NIS and NRPS proteins has been documented [24,34,37].

Siderophore biosynthesis is tightly linked to iron acquisition as microorganisms produce these molecules in response to iron scarcity. Understanding the iron acquisition mechanism, specifically siderophore secretion and iron uptake, is crucial for comprehending siderophore biosynthesis pathways. This knowledge enables researchers to gain insights into how microorganisms adapt to iron deficiency and exploit these pathways to thrive in competitive and nutrient-limited environments.

## 3. Iron Acquisition

**Figure 7:**
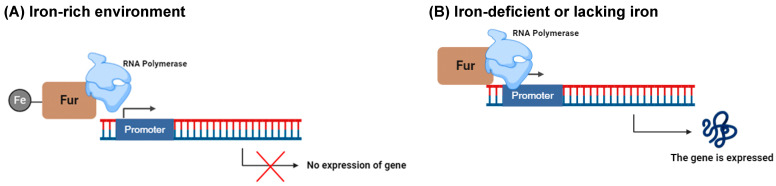
(**A**) In this model, excess iron forms a complex with the Fur box protein at the promoter, blocking DNA and RNA polymerases and inhibiting gene expression. (**B**) Conversely, when iron levels are low, Fur proteins dissociate from RNA polymerases, facilitating gene expression (adapted from [25], created in BioRender).

Microorganisms must precisely regulate the enzymes and transport systems responsible for the coordinated processes of siderophore production, secretion, iron uptake via siderophores, and iron release. The activation of iron acquisition systems occurs primarily in environments where iron reserves are scarce [43]. The levels of iron within cells trigger responses through the ferric uptake regulator (Fur), which serves as the central transcription factor governing iron balance across various bacterial species. When cellular iron levels are sufficient, the formation of the Fur-Fe^2+^ complex represses the transcription of genes associated with iron uptake, thereby preventing an excessive influx of iron into the cell (Figure 7). Conversely, in conditions where iron is limited, apo-Fur predominates, leading to the activation of genes involved in iron acquisition and facilitating the transport of iron into the cell (Figure 7) [44].

Bacterial and fungi strategies for acquiring iron encompass various mechanisms, including siderophore-mediated transportation, direct uptake via divalent metal transporters, or directly seizing iron from host proteins [25,27].

### 3.1. Siderophore Secretion

**Figure 8:**
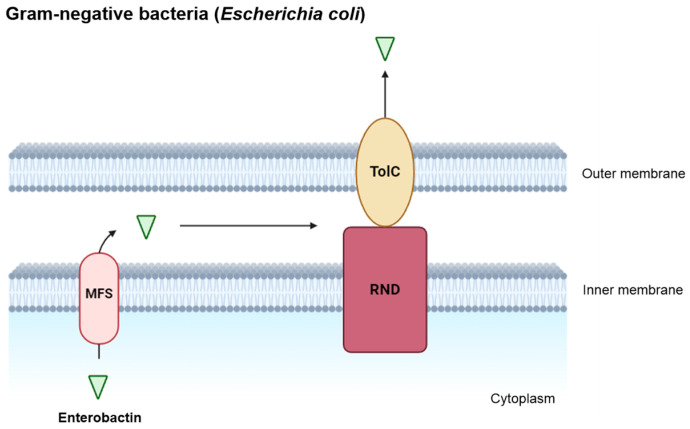
Secretion of enterobactin in Gram-negative bacteria, facilitated by MFS proteins and involving the participation of a TolC complex along with an associated resistance/nodulation/cell division (RND) efflux pump (adapted from [48], created in BioRender).

The release of siderophores requires the activity of at least one of the subsequent efflux pump families: major facilitator superfamily (MFS), ATP-binding cassette (ABC), or the Gram-negative-specific resistance/nodulation/cell division (RND) [22]. The MFS, a notably vast and varied superfamily, is responsible for facilitating uniport, solute–cation symport (utilizing protons or sodium), and/or solute–proton or solute–solute antiport [24,40]. These transporters demonstrate specificity towards a wide array of compounds, encompassing primary metabolites, pharmaceuticals, neurotransmitters, and siderophores (for efflux), as well as organic and inorganic anions. The pathway utilised by enterobactin (Figure 1) in *Escherichia coli* is arguably one of the most extensively researched routes for siderophore secretion, comprising multiple steps [45]. Initially, enterobactin is moved from the cytoplasm to the periplasm by an MFS-class efflux pump [46]. Subsequently, a coordinated effort between an inner-membrane RND-class efflux pump and the outer-membrane protein channel TolC facilitates the transportation of enterobactin across the outer membrane (Figure 8) [47].

Members of various families within the RND group collaborate with a membrane fusion protein (MFP) and an outer-membrane factor (OMF) to facilitate efflux across both membranes of the Gram-negative bacterial cell envelope, accomplished in a single energy-coupled process [40,49]. The ABC-type transporter superfamily encompasses both efflux and uptake transport systems, typically facilitating transport through ATP hydrolysis without protein phosphorylation. These transporters comprise two integral membrane domains and two cytoplasmic domains (ABC subunits), which bind and hydrolyse nucleoside triphosphate (NTP), typically ATP [50].

### 3.2. Cellular Transport Systems for Uptake of Siderophore-Delivered Iron in Bacteria and Fungi

The mechanism of iron-loaded siderophore uptake varies between Gram-negative and Gram-positive bacteria, primarily due to the absence of an outer membrane in Gram-positive bacteria, which eliminates the need to translocate iron-loaded siderophores across it [51]. Given that eukaryotes lack ABC-type uptake systems, the mechanism of Fe–siderophore uptake in fungi differs somewhat from that in bacteria [27]. Typically, MFS-type transporters are responsible for importing full Fe–siderophore complexes [40]. In contrast, Gram-negative bacteria utilize specific outer-membrane (OM) receptors to uptake ferrisiderophore complexes (Fe^3+^ bound to siderophores form complexes that exceed the size limit (600 Da) for passage through porins, which typically permit smaller solutes to permeate the outer membranes of Gram-negative bacteria), a process facilitated by the energy-transducing TonB-ExbB-ExbD system, driven by the cytosolic membrane (CM) potential. TonB, a 26 kDa protein, can be described as a protein with three domains [52,53]. When a siderophore binds to its TonB-dependent transporter (TBDT), it initiates a signal transmission through the outer membrane, leading to a disruption (referred to as unfolding or undocking) of the TonB box. Although the exact nature of this transmitted signal remains somewhat unclear, in certain TBDTs, it seems to entail significant conformational alterations in extracellular loops. These loops fold inward upon siderophore binding, enclosing the ligand and introducing new residues to the binding site [53].

Outer-membrane siderophore receptors are constructed as β-barrel proteins comprising a 22-stranded tube, which spans the outer membrane, facilitating the passage of siderophore complexes into the periplasm. Furthermore, OMs are typically induced in response to iron deprivation and are typically absent under conditions of sufficient iron availability [54]. Transport across the initial membrane occurs through TBDT, while transport across the inner membrane involves ABC transporters coupled with periplasmic binding proteins (PBPs) or permeases dependent on proton motive force. In the cytosol, these complexes are likely dissociated through reduction mechanisms [7].

**Figure 9:**
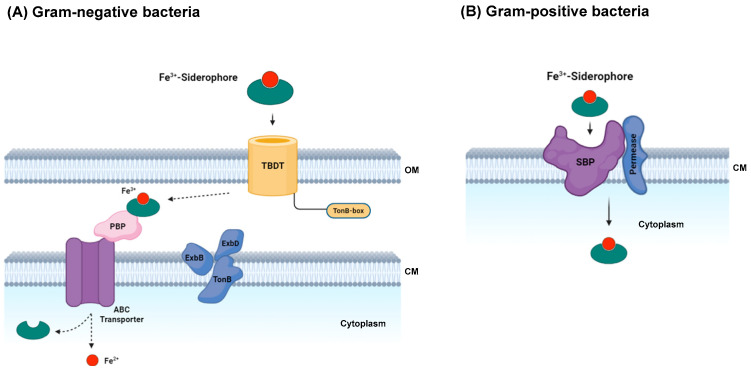
Siderophore import mechanisms. (**A**) Process of siderophore transport in Gram-negative bacteria, ferric siderophore transport relies on the protonmotive force of the inner membrane, mediated by the TonB complex (blue). TonB interacts with outer-membrane transporters (TBDT) through the TonB-box motif (yellow). Inner-membrane transport requires a periplasmic binding protein (pink) and an ABC transporter (purple). (**B**) Process of siderophore transport in Gram-positive bacteria; the import of siderophores includes recognition by a SBP situated on the cell membrane (purple). An associated permease (blue) is tasked with the transport of ferric siderophores across the membrane (adapted from [48], created in BioRender).

Upon internalization, the ferrisiderophore complex undergoes dissociation to release the bound iron through one of two cellular metabolic processes. The predominant method involves the reduction of ferric iron to its ferrous state, facilitated by non-specific ferrisiderophore reductases, commonly flavin reductases with multifunctional roles within the cell. This reduction decreases the affinity of the siderophore for ferrous iron, facilitating the spontaneous release of iron. Alternatively, the second mechanism relies on the enzymatic hydrolysis of the siderophore, specifically targeting its bonds to weaken interactions with iron, thereby enabling liberation (Figure 9) [26].

The process of siderophore transport in Gram-positive bacteria closely resembles that in Gram-negative bacteria, wherein ABC transporters facilitate translocation into the cytoplasm. Gram-positive bacteria, having only one membrane, feature a less complex siderophore uptake system. Typically, they express siderophore-binding protein (SBP) paired with a permease. When SBP binds to an extracellular ferric siderophore, it induces a conformational change in the SBP–permease complex, facilitating the transport of the ferric siderophore across the membrane and into the cytoplasm (Figure 9) [26].

Iron acquisition in microorganisms is closely tied to siderophore biosynthesis, with both processes playing a key role in regulating cellular iron levels. Targeting and inhibiting siderophore biosynthesis have emerged as a potential strategy for controlling microbial growth. By interfering with the enzymes responsible for siderophore production, the pathogen’s ability to acquire iron can be hindered, limiting its growth and spread.

## 4. Inhibition of Siderophore Biosynthesis

Microbial siderophores represent a crucial iron acquisition system, making them attractive targets for antimicrobial intervention. By disrupting siderophore biosynthesis, researchers aim to create bacteriostatic conditions that limit pathogen proliferation. Recent advances in crystallography and rational drug design have propelled this field forward, offering novel inhibitors with potential therapeutic applications. However, while these developments show promise, several challenges remain, including bacterial resistance and selectivity issues. Below, we explore key strategies and notable discoveries in siderophore biosynthesis inhibition while synthesizing their implications for drug development [40].

### 4.1. Inhibitors Targeting NRPS Biosynthesis

Research on inhibitors targeting NRPS-based biosynthesis has primarily focused on two critical enzymatic processes: the activation of amino acids through adenylation, and the formation of the thiol tether on the carrier domain [40]. NRPSs operate through a modular architecture, with each module dedicated to incorporating a specific amino acid into the growing peptide chain. In some fungi, all the necessary modules are encoded within a single gene, producing one massive, multimodular protein. However, more commonly, NRPS pathways involve multiple large proteins that work together through intricate intra- and intermolecular interactions to synthesize the peptide [55,56].

In addition to the core catalytic domains, NRPSs often include auxiliary domains that introduce structural diversity. Some of the most common modifications include methylation via S-adenosylmethionine (SAM)-dependent methyltransferases, epimerization, and the heterocyclization of serine, threonine, and cysteine residues to form oxazoline and thiazoline rings. These modifications can enhance the stability and bioactivity of the peptide. Interestingly, not all NRPSs terminate with the usual thioesterase domain; some instead feature a reductase domain, which catalyses the NADPH-dependent formation of a terminal aldehyde. This aldehyde can then undergo further modifications, either spontaneously or through enzymatic catalysis, leading to an even wider range of structural diversity [57,58,59].

#### 4.1.1. Phosphopantetheinyl Transferase Inhibitors

**Figure 10:**
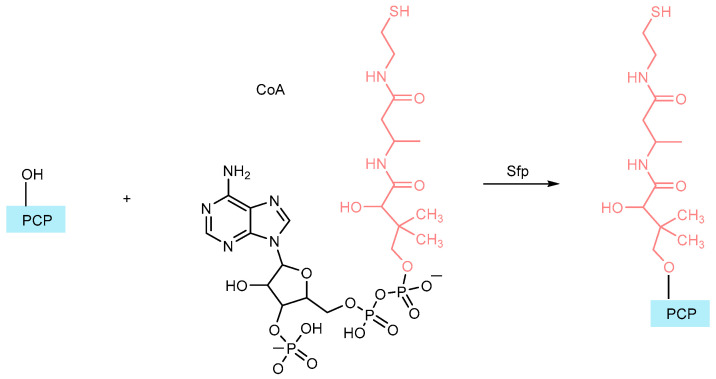
The post-translational modification of carrier domains results in the creation of a phosphopantetheinyl swinging arm. The phosphopantetheine group of CoA is highlighted in red. PCP refers to the peptidyl carrier protein. Sfp is a versatile PPTase from *Bacillus subtilis* frequently used to conduct this reaction in vitro (adapted from [34]).

Each standard NRPS module features a peptidyl carrier protein (PCP), a small (8 kDa) domain structurally similar to the acyl carrier proteins (ACPs) involved in fatty acid transport and synthesis [60]. Before the PCPs can participate in NRPS and PKS siderophore biosynthesis, they must undergo a crucial post-translational modification. Specifically, PCP domains possess a conserved serine residue that is modified by phosphopantetheinyl transferase (PPTase), which attaches a phosphopantetheine cofactor derived from Coenzyme A (CoA). This modification creates a “swinging arm”, essential for peptide chain elongation (Figure 10) [61,62].

**Figure 11:**
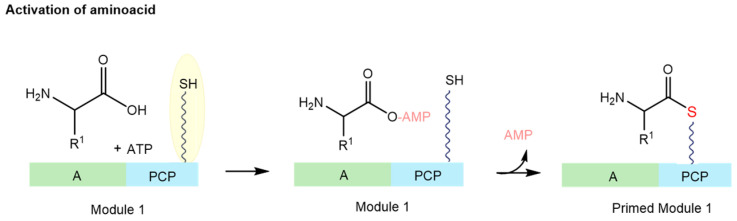
Initiation phase of NRPS biosynthesis. Green: adenylation domain (A); blue: PCP (peptidyl carrier protein) (adapted from [34]).

In NRPS biosynthesis, amino acids are covalently attached via thiol groups, allowing for chain elongation and subsequent modifications. This attachment process is depicted in Figure 11, with the thiol connections highlighted in yellow. PPTases, particularly the Sfp type, play a central role in modifying the carrier domains of NRPS and PKS enzymes and have, therefore, become important targets for inhibition. One widely used approach to measure PPTase enzyme activity is through radiolabel incorporation, tracking the addition of tritiated-CoA to a carrier domain [61,62,63].

**Figure 12:**
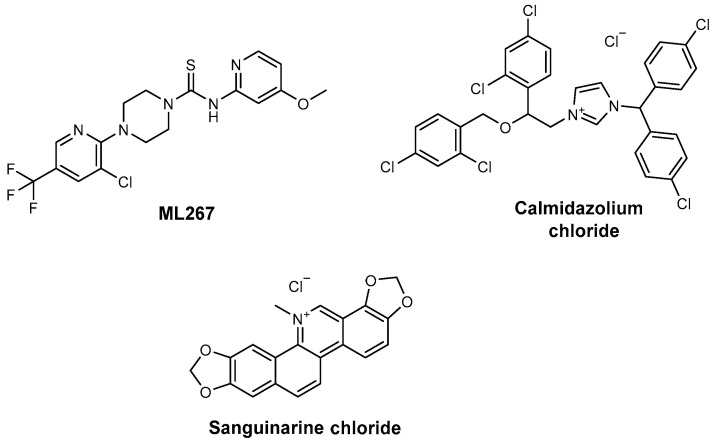
Sfp-PPTase inhibitor ML267, calmidazolium chloride, and sanguinarine chloride.

A high-throughput screening of over 300,000 compounds led to the discovery of ML267 (Figure 12) as a selective inhibitor of Sfp-PPTase [64]. ML267 functions as an allosteric, reversible inhibitor with a high nanomolar IC_50_ value and has demonstrated bactericidal activity against Gram-positive pathogens, including *B. subtilis* and both methicillin-sensitive and methicillin-resistant strains of *Staphylococcus aureus* [65,66]. Studies suggest that ML267 specifically inhibits *B. subtilis* Sfp-PPTase (IC_50_ = 0.29 μm) and AcpS-PPTase (IC_50_ = 8.1 μm), without affecting the corresponding human orthologue (IC_50_ > 114 μm), underscoring its antibacterial properties [67,68]. ML267 underwent safety and pharmacological evaluation, showing promising characteristics for therapeutic development. Cytotoxicity tests in human HepG2 liver cells showed no significant effects, and in vivo studies revealed no signs of acute toxicity. Additionally, ML267 exhibited minimal interaction with human glutathione S-transferase A1-1 (GST A1-1), addressing concerns about cross-reactivity often associated with thiol-sensitive compounds. In this study, in addition to the detailed molecular profile of ML267, researchers have unveiled the mechanism of action; the in vitro absorption, distribution, metabolism, and excretion; and the in vivo pharmacokinetic profiles to further demonstrate the potential utility of this small-molecule inhibitor [68]. Pharmacological profiling demonstrated favourable drug-like properties, including optimal absorption parameters, metabolic stability, and excretion characteristics, in preliminary in vitro ADME studies. Subsequent in vivo pharmacokinetic studies showed adequate plasma concentration levels, a half-life of 2.4 h via intravenous (IV) administration and 2.0 h via intraperitoneal (IP) administration, and suitable bioavailability (after IP administration was 98.5%), supporting its potential utility for testing in infection models targeting bacterial pathogenesis mechanisms. These findings highlight ML267 as a promising candidate for the advanced preclinical investigation of antimicrobial strategies.

In a separate screening,13 small molecules were assayed against PptT for inhibition in a fluorescence polarization assay with both VibB from *Vibrio cholerae* and mycocerosic acid synthase (MAS) from *Mycobacterium tuberculosis* as the target carrier proteins [69]. Two promising inhibitors, calmidazolium chloride [IC_50_ = 2.0 μM (VibB) and IC_50_ = 4.9 μM (MAS)] and sanguinarine chloride [IC_50_ = 22 μM (VibB) and IC_50_ = 4.9 μM (MAS)] (Figure 12), were identified, both exhibiting low micromolar IC_50_ values [69]. Notably, sanguinarine also inhibits ornithine hydroxylase, despite no apparent structural or functional similarities between the two target enzymes [69,70].

**Figure 13:**
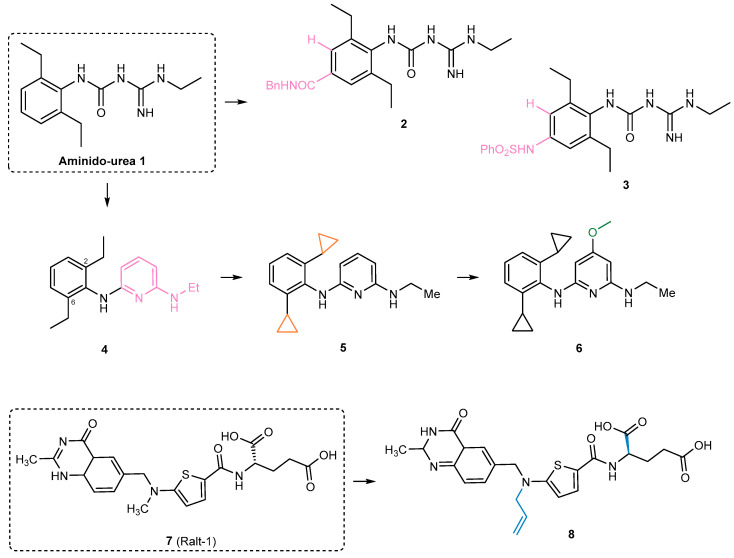
PptT inhibitors aminido-urea **1** and optimized **2** (*p*-benzamide), **3** (*p*-phenylsulfonamide), **4, 5** and **6** derivatives; Ralt-1 (**7**) and its optimized analogue **8**. Colours indicate structural modifications.

The inhibitory properties of aminidourea 1-[(2,6-diethylphenyl)-3-*N*-ethylcarbamimodoyl]urea (**1**) (Figure 13) were evaluated against phosphopantetheinyl transferases PptAb from *Mycobacterium abscessus* and PcpS from *Pseudomonas aeruginosa*, two pathogens linked to cystic fibrosis and bronchiectasis, respectively [71]. In vitro analysis demonstrated that **1** effectively inhibits PptAb at nanomolar concentrations, reaching full inhibition at micromolar levels (IC_50_ = 2.3 μM) [71]. However, there was no significant inhibition of PcpS, even at high concentrations of **1**, consistent with prior studies that reported its ineffectiveness against *P. aeruginosa* [71,72,73]. Despite its limited antimicrobial efficacy in broth culture against *M. abscessus* and *P. aeruginosa* [74], subsequent studies revealed promising results for **1**, including its ability to eradicate *M. tuberculosis* in murine models and its in vitro activity against *Mycobacterium smegmatis* and *Mycobacterium bovis* Bacillus Calmette-Guerin (BCG) [75]. Identified as a PptT inhibitor (IC_50_ = 2.3 μM) with both in vitro and in vivo activity against *M. tuberculosis*, **1** is now considered a potential candidate for tuberculosis treatment development [76]. While lacking broad-spectrum activity against other bacterial species, yeasts, or animal cells, its specificity makes it a molecule of scientific interest [73]. Efforts to refine its pharmacological properties have led to the development of *para*-substituted analogues, such as **2** (*p*-benzamide) and **3** (*p*-phenylsulfonamide) (Figure 13), which retain comparable potency (IC_50_ = 2.5 μM and IC_50_ = 3.8 μM, respectively) while exhibiting reduced activity on ion channels associated with cardiac toxicity [74,75]. These new derivatives suggest a promising direction for developing safer and more effective tuberculosis treatments. In another investigation, compound **4** (Figure 13), which showed significant structural similarity to **1**, demonstrated promising inhibition of PptT, with an IC_50_ of 0.71 μM (FP assay) and a moderate MIC_90_ of 42 μM [73]. Subsequent modifications involved altering the ethyl groups at the 2 and 6 positions of the aniline in **4**. Replacing these ethyl groups with isopropyl led to a decrease in both whole-cell and enzymatic activity, contrary to the observed trend in **1** analogue, where ortho-substituted branching was beneficial [73]. However, compound **5** (Figure 13), featuring cyclopropyl groups, maintained some enzymatic activity and remained promising with an IC_50_ of 8.4 μM (BpsA assay) while improving on target potency (MIC_90_ = 22 μM). Further exploration involved substituting the pyridine ring, with compound **6** (Figure 13) emerging as the most potent (IC_50_ = 0.72 μM, BpsA assay), exhibiting an MIC_90_ of 16 μM [73]. Finally, compounds **4** and **6** underwent evaluation for potential cardiotoxicity, revealing no instances of a compound within this new class, combining outstanding potency with a favourable side effect profile [73]. Notably, one of the most potent (IC_50_ = 65 nM) PptT inhibitors identified to date is raltitrexed (Ralt-1) (**7**, Figure 13), a thymidylate synthase inhibitor marketed as Tomudex for cancer treatment [77]. Further, **7** exhibited a normalized maximum inhibition exceeding 100%, surpassing the positive control, amidinourea **1** (10 μM). However, it also displayed limitations, including anti-proliferative effects on human cells and a lack of whole-cell activity against *M. tuberculosis* [78]. While amidinoureas inhibited PptT noncompetitively, never exceeding 90% inhibition, **7** demonstrated superior potency. Modifications to its structure, such as replacing the nitrogen with an allyl moiety, led to the development of **8**, a more potent analogue (IC_50_ = 18 nM) with enhanced interaction with the hydrophobic pocket, which showed no activity against *M. tuberculosis* in whole-cell assays (Figure 13) [78]. The absence of on-target activity in whole cells of Ralt-1 (**7**) and its analogue (**8**) can be attributed to a combination of factors: limited cellular uptake, active efflux, and rapid metabolism.

**Figure 14:**
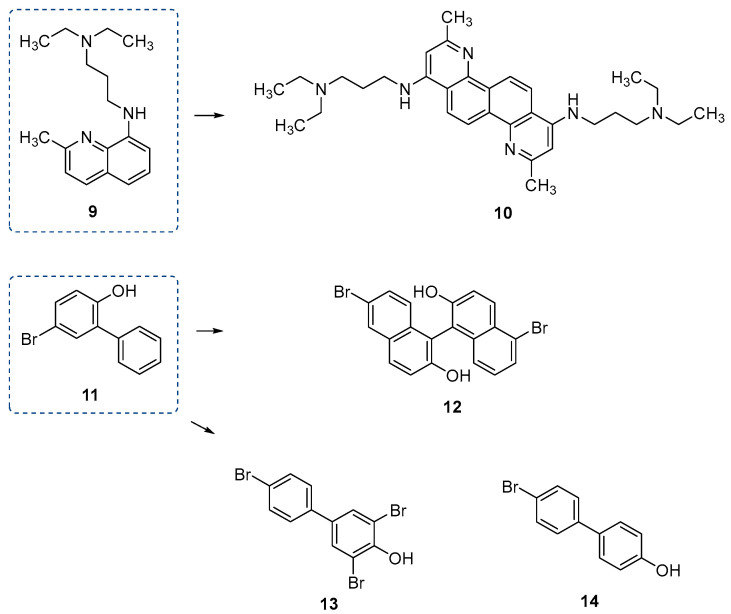
PptT inhibitors: **9** and its optimized analogue **10**; **11** and its optimized analogues **12**–**14**.

In another study, a series of novel compounds were screened for inhibitory activity against PptT, a critical drug target in *M. tuberculosis*. Researchers identified *N,N*-diethyl-*N’*-(2-methylquinolin-8-yl)propane-1,3-diamine (**9**) as a highly effective inhibitory scaffold presenting an IC_50_ of 2.25 μg/mL (8.29 μM) [79]. Several analogues of compound **9** (Figure 14), which contains this scaffold, were tested, with five displaying IC_50_ values ≤ 1 μg/mL. Among them, **10** (Figure 14) showed the most promising activity, achieving an IC_50_ of 0.25 μg/mL [79]. Structural analysis of **9** and its analogues highlighted the importance of the quinoline moiety linked to an amino group and an extended carbon chain in achieving strong inhibitory effects. Further molecular studies provided insights into how these inhibitors interact with the PptT active site. Predicted interactions revealed key binding elements, including hydrogen bonds and salt bridges involving Glu 157 and the amino groups in the side chains, as well as interactions with the phenyl group. These structural insights explain the strong inhibitory effects observed in enzymatic assays. Notably, **10** not only exhibited potent enzyme inhibition [IC_50_ = 0.25 μg/mL (0.49 μM)] but also demonstrated strong antimycobacterial activity, with an MIC_90_ of 10 μg/mL against *M. tuberculosis* in broth culture, outperforming its parent molecule, **9**. This positions **10** as a promising candidate to further lead optimization in tuberculosis drug development [79]. Additionally, researchers identified another inhibitory scaffold associated with compound **11** [IC_50_ = 43.16 μg/mL (173.16 μM)], featuring a 5-bromobiphenyl-2-ol core structure. Several analogues of **11** demonstrated enzyme inhibitory activity, with **12**–**14** exhibiting IC_50_ values of 4.12 μg/mL (9.28 μM), 5.0 μg/mL (12.29 μM), and 7.12 μg/mL (28.58 μM), respectively (Figure 14). By combining both in silico and in vitro methodologies, this study provides a strong foundation for designing next-generation inhibitors targeting *M. tuberculosis* [79].

#### 4.1.2. Inhibitors of the Adenylation Domain

**Figure 15:**
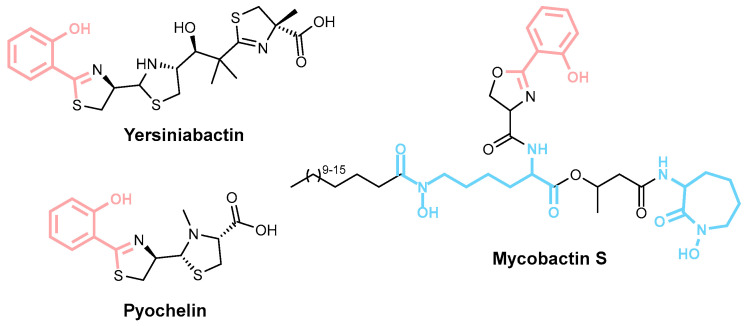
Salicylate-capped siderophores yersiniabactin, mycobactin S, and pyochelin. Salicylate caps are depicted in coral, and hydroxylysine residues in blue.

**Salicylate adenylation inhibitors.** The first step in the NRPS biosynthetic pathway involves priming carrier domains with either initiator or elongation units. This process is carried out by adenylation domains, which can be embedded within the NRPS enzyme complex or exist as standalone domains. A major area of interest has been adenylation domains responsible for activating the hydroxy acid salicylate, a crucial step in the synthesis of salicylate-capped siderophores. These siderophores, including yersiniabactin, mycobactin, and pyochelin (Figure 15), play essential roles in bacterial acquisition [40,80].

**Figure 16:**
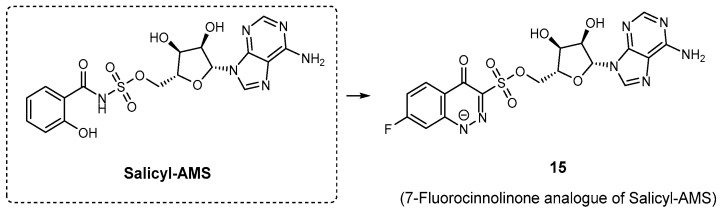
Salicylate adenylation inhibitors: Salicyl-AMS and its 7-fluorocinnolinone analogue **15**.

Given their importance, potential inhibitors have been designated as bisubstrate analogues that incorporate modified aryl acid and adenosine components, linked by a nonhydrolyzable linker to maximize structural diversity. The first notable analogue identified as an inhibitor of aryl acid A domains was 5’-O-[*N*-(salicyl)-sulfamoyl] adenosine (Salicyl-AMS, Figure 16) [81]. This molecule is classified as a bisubstrate inhibitor because it bridges the salicylate ring with the sugar nucleotide. Salicyl-AMS exhibited potent inhibitory activity, competing with ATP but not with salicylate, indicating that it binds to the same enzyme form as ATP and interferes with the initial adenylation step. This nonhydrolyzable intermediate demonstrated strong inhibition, with IC_50_ values against salicylate adenylation enzymes such as MbtA (*M. tuberculosis*), YbtE (*Yersinia pestis*), and PchD (*P. aeruginosa*), with an IC_50_ of 10.7, 14.7, and 12.5 μM, respectively [70]. Additionally, Salicyl-AMS showed low micromolar EC_50_ values for inhibiting the growth of *M. tuberculosis* and *Y. pestis* in iron-depleted conditions, making it a promising candidate for targeting siderophore biosynthesis. In another study, six synthetic acyl analogues, among them salicyl-AMS, and five new analogues representing either 5’-*O*-[*N*-(aryl)-sulfamoyl]adenosine (AMS) or 5’-*O*-[*N*-(aryl)-hydroxamoyl] adenosine (AMN) derivatives were screened for inhibitory effects of the DHB-activating adenilation domain of *Bacillus subtilis*, DhbE [82]. Four AMS compounds exhibited strong inhibition, ranging from 86 to >99% loss of DhbE activity, with salicyl-AMS inhibiting 99.6% of the DhbE activity [82]. Despite its potent inhibitory capacity, salicyl-AMS suffers from nonoptimal drug disposition and pharmacokinetic (PK) properties, in part caused by the ionized acyl-sulfamate, and the possible hydrolysis across the acyl-sulfamate linkage would liberate cytotoxic 5′-*O*-(sufamoyl)adenosine [83]. Additionally, PK studies in mice revealed detectable levels of salicyl-AMS in plasma and lung tissue after oral and intraperitoneal administration, although oral bioavailability was limited (Cmax = 1.2 μg/mL, AUC = 58.6 μg·min/mL) [84]. Despite rapid clearance from the lungs (half-lives of 13.3–19.3 min), the drug demonstrated in vivo efficacy. Furthermore, salicyl-AMS exhibited no cytotoxicity against P388, HepG2, and Vero cell lines, with CC_50_ values exceeding 200 μM. Even with this favorable in vitro safety profile, the preclinical advancement of Salicyl-AMS was hindered by a short pharmacokinetic half-life and toxicity issues observed in vivo, which limited its dosing potential [85]. With this in mind, Dawadi and co-workers have designed and synthesized structural analogues of Salicyl-AMS, particularly in the context of *M. tuberculosis*, without this potential liability. Replacement of the aromatic hydroxyl group of the salicylate residue with a fluor-substituted cinnolone moiety [83], difluorination of the ribose group [86], and the incorporation of bulky phenyl rings at the adenine unit [81] led to improvements in the pharmacokinetic profile. Among these, the 7-fluorocinnolinone analogue (**15**, Figure 16) emerged as the most promising candidate [83]. In this compound, part of the acyl sulfamate linker was replaced by a heterocycle, significantly enhancing its stability and potency [85]. **15** demonstrated low nanomolar inhibition of the mycobacterial salicylate ligase MbtA (apparent *K*i = 12 nM) and exhibited excellent antimycobacterial activity under iron-deficient conditions (MIC = 2.3 μM), highlighting its potential as a lead compound for further drug development [83].

**Figure 17:**
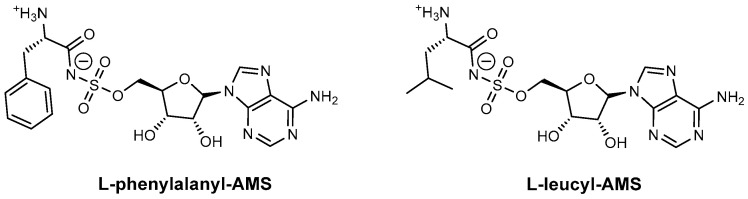
Aminoacyl-AMS inhibitors: L-phenylalanyl-AMS and L-leucyl-AMS.

**Aminoacyl-AMS linear inhibitors.** L-phenylalanyl-AMS (Figure 17), and L-leucyl-AMS (Figure 17) are structural analogues designed to specifically inhibit adenylation domains in NRPS, by mimicking their natural substrates. Phenylalanyl-AMS selectively inhibits the gramicidin S synthetase A subunit (GrsA) phenylalanine adenylation domain (PheA) (*Bacillus brevis*), while leucyl-AMS the surfactin synthetase C subunit (SrfA-C) leucine adenylation domain (LeuA) (*B. subtilis*) [87]. Both compounds exhibit nanomolar Ki values and also inhibit tRNA synthetases (Figure 17) [85,87].

**Figure 18:**
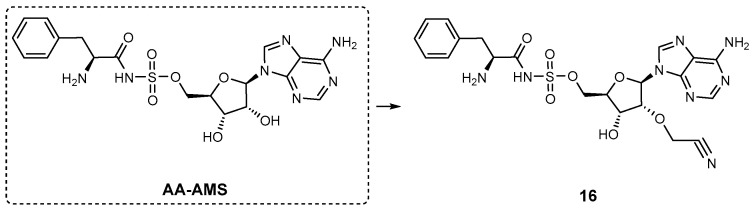
5’-O-*N*-(aminoacyl or arylacyl)sulfamoyladenosine (**AA-AMS**) and its optimized analogue **16**.

Another class of inhibitors, 5’-*O*-*N*-(aminoacyl or arylacyl)sulfamoyladenosine (AA-AMS, Figure 18), was developed to target the amino acid-activating A-domains of NRPS [88]. AA-AMS functions as a bisubstrate, tight-binding small-molecule inhibitor [89]. However, its practical application is limited by poor bacterial cell permeability due to its high hydrophilicity. To overcome this limitation, researchers investigated modifications at the 2′-OH position of the AMS scaffold, introducing various functional groups to enhance both enzyme binding and cellular uptake. Among these, a cyanomethyl substitution at the 2′-OH position (**16**, Figure 18), exhibited significant inhibitory activity against both recombinant and intracellular gramicidin S synthetase A (GrsA) in the gramicidin S-producing bacterium *Aneurinibacillus migulanus* ATCC 9999 [87,88].

**Figure 19:**
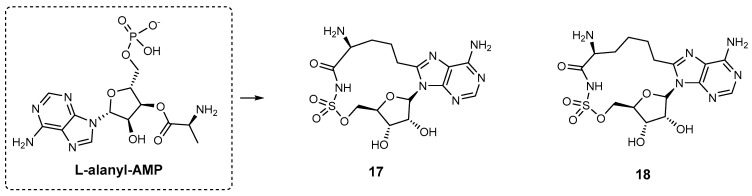
L-alanyl-AMP and its optimized analogues **17** and **18**.

**Aminoacyl-AMS macrocyclic inhibitors.** Tan and co-workers investigated selective NRPS inhibitors and identified a class of aminoacyl-AMS (sulfamoyladenosine) macrocycles that specifically target amino acid adenylation domains in NRPS [90,91]. Macrocycles **17** and **18** (Figure 19 were developed as constrained analogues of L-alanyl-AMP, showing strong inhibitory activity in cysteine adenylation of HMWP2 (high molecular weight protein 2), a component of the *Y. pestis* yersiniabactin siderophore synthetase. Compound **17** (cyclo^8C2^‚-L-Ala-AMS, *K*i = 1.7 μM) demonstrated greater potency than **18** (cyclo^8C3^‚-L-Ala-AMS, *K*i = 5.4 μM). Interestingly, the two-carbon-linked macrocycle **17** was slightly more potent than L-alanyl-AMS (Figure 19). Neither **17** nor **18** exhibited inhibitory activity against aminoacyl-tRNA synthetases at concentrations up to 250 μM [91]. However, the authors found that although **17** was a potent biochemical inhibitor of the cysteine adenylation domain, it did not inhibit yersiniabactin production in *Y. pestis* cell culture.

**Figure 20:**
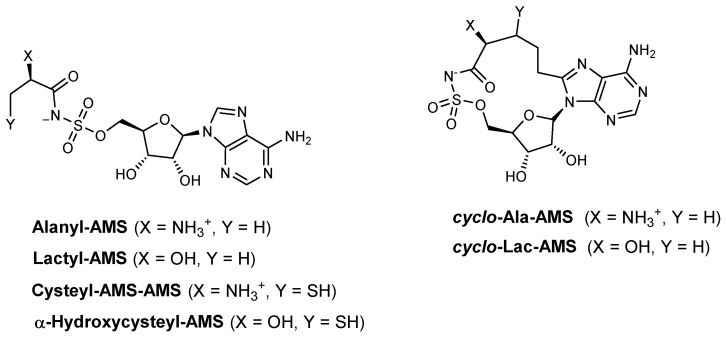
Aminoacyl-AMS macrocyclic inhibitors.

To improve the cell permeability of these inhibitors, the authors synthesized a series of acyl-AMS analogues to systematically assess the impacts of the α-amino-to-α-hydroxy modification, the amino acid side chain, and the macrocyclic constraint upon inhibition of the cysteine adenylation domain of HMWP2 [90]. To determine the non-selective inhibitory activity of linear and cyclic compounds against both the Cys adenylation domain of *Y. pestis* HMWP2 and aminoacyl-tRNA synthetases (IVT), the authors initially used the ATP–^32^PPi isotope exchange assay [91]. One of the cyclic compounds, 5′-*O*-[*N*-aminoacyl-sulfamoyl]-adenosine (Cyclo-Ala-AMS) (Figure 20), was designed as a selective inhibitor of these domains. Unlike its linear aminoacyl-AMS analogue [*K*i ^app^ (HMWP2) = 2.5 μM, IC_50_ (IVT) = 0.16 μM], Cyclo-Ala-AMS was not able to inhibit aminoacyl-tRNA synthetases [*K*i ^app^ (HMWP2) = 1.7 μM, IC_50_ (IVT) = >250 μM] [90]. This selectively arises from the macrocyclic conformation, which promotes or enforces the cisoid conformation, making the molecule non-hydrolysable and selectively inhibiting NRPS adenylation domains without affecting aminoacyl-tRNA synthetases [91]. Although this macrocycle demonstrated potent biochemical inhibition of HMWP2 (high-molecular-weight protein 2), a component of the *Y. pestis* yersiniabactin siderophore synthetase, it failed to inhibit yersiniabactin production in *Y. pestis* cell culture [85,90].

**Figure 21:**
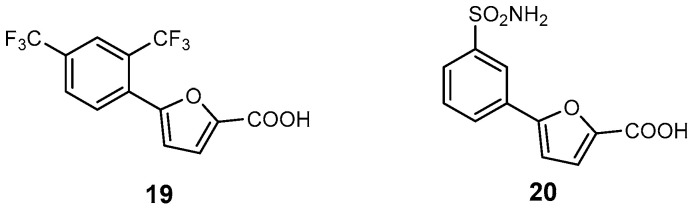
Inhibitors of salicylate synthase: 5-(2,4-bis(trifluoromethyl)phenyl)furan-2-carboxylic acid (**19**) and 5-(3-sulfamoylphenyl)furan-2-carboxylic acid (**20**).

**Salicylate synthase inhibitors.** Siderophore biosynthesis has been extensively studied in *M. tuberculosis* but has received less attention in non-tuberculous mycobacteria (NTM) [92]. The first enzyme in this pathway, salicylate synthase (SaS), known as MbtI in Mtb, catalyses the conversion of chorismic acid to salicylic acid [29,92,93]. A recent study focused on Mab-SaS, the first enzyme involved in siderophore biosynthesis in NTMs. A series of inhibitors were identified based on the 5-phenylfuran-2-carboxylic acid scaffold [94]. Structural analysis revealed how these inhibitors interact with the enzyme’s binding pocket. Among them, compound **19** emerged as a potent inhibitor with an IC_50_ of 5.3 μM (**19,** Figure 21) [29]. It is worth noting that the low toxicity of this compound has been previously evaluated in human MRC-5 fibroblasts and blood cells [94]. Further exploration of a focused library of *m*-substituted 5-phenylfuran-2-carboxylic acid derivatives led to the discovery of a more effective inhibitor, compound **20**, which exhibited an improved IC_50_ of 2.6 μM against Mab-SaS (**20,** Figure 21) [94]. Thus, compound **20** was subjected to further analysis to ensure it did not act as a PAIN compound, and the tests confirmed that it did not exhibit such behaviour. As expected, **20** showed no significant cytotoxic effects, with 99.5% of cells remaining viable at the highest tested concentration (10 μM, approximately 5-times the IC_50_) [94]. It is important to note that cardiotoxicity, typically caused by off-target interactions with the hERG potassium channel, is widely recognized as a common side effect of drugs in clinical phases or post-market surveillance. Notably, none of the Mab-SaS inhibitors tested were predicted to block the hERG channel [29,94].

**Figure 22:**
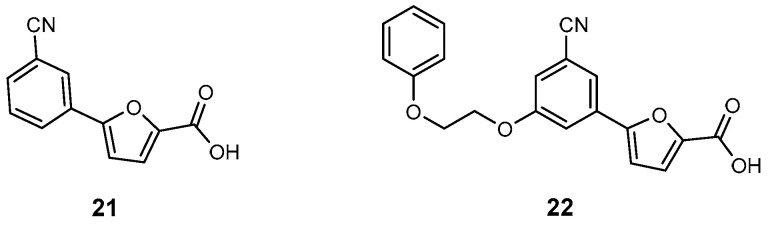
Inhibitors of salicylate synthase: 5-(3-cyanophenyl)furan-2-carboxylic acid (**21**) and its optimized analogue **22**.

In another study, researchers synthesized and tested a series of analogues of 5-(3-cyanophenyl)furan-2-carboxylic acid (**21**, Figure 22), the most potent MbtI competitive inhibitor identified to date (*m*-cyano compound, IC_50_ = 6.3 μM and Ki = 4 μM). This compound also demonstrated strong activity against mycobacterial cultures [95]. The study aimed to elucidate the key structural features required for effective MbtI inhibition and strong antitubercular activity. Among the newly identified inhibitors, compound **22** (Figure 22) showed significant potency against MbtI (IC_50_ = 12.1 μM), exhibited no cytotoxicity toward human cells, and demonstrated enhanced in vitro efficacy against mycobacterial growth (MIC_99_ = 63 μM) under iron-limited conditions [96].

Most MbtI inhibitors discovered so far contain a common carboxylate motif, which is believed to contribute to metal ion sequestration within the active site. However, this interaction may not be essential for ligand binding or inhibition. Biological studies on a derivative in which the carboxylate pharmacophore motif was replaced with an amide group demonstrated strong inhibitory activity, suggesting that metal chelation should not be considered a mandatory feature for developing MbtI inhibitors [95].

**Figure 23:**
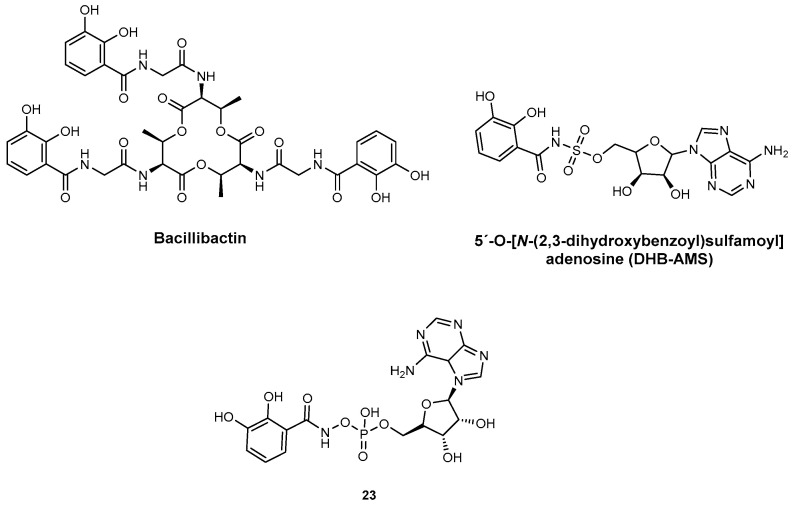
Structures of Bacillibactin and dihydroxybenzolate adenylation inhibitors DHB-AMS and hydroxyamoyl phosphate compound **23**.

**Dihydroxybenzolate adenylation inhibitors.** Inhibitors have also been developed to target dihydroxybenzoate (DHB)-adenylating enzymes, which play a crucial role in the biosynthesis of DHB-capped siderophores. These siderophores are produced by various bacteria, including *E. coli* (enterobactin) (Figure 1), *Vibrio cholerae* (vibriobactin) (Figure 1), and *B. subtilis* (bacillibactin) (Figure 23) [40,80]. One study focused on inhibiting dihydroxybenzoate-AMP ligase (EntE) from *E. coli*, using an adenylate analogue, 5′-O-[*N*-(2,3-dihydroxybenzoyl)sulfamoyl]adenosine (DHB-AMS, Figure 23). DHB-AMS exhibited highly potent inhibition, with low picomolar values for both inhibition and dissociation constants. Since this biosynthetic pathway is absent in mammals, antibiotics targeting this enzyme are expected to be highly selective to bacteria, potentially reducing off-target effects on human hosts [97]. In a separate study, DHB-AMS was selected for detailed inhibition analysis, focusing on the enzymes *B. subtilis* DhbE and YbtE of *Y. pestis* [82]. The inhibition constants (*K*i = 85 nM for DhbE and *K*i = 54 nM for YbtE) demonstrate effective inhibition of the A domain by DHB-AMS [82]. A hydroxyamoyl phosphate compound (**23,** Figure 23), a DHB-containing analogue, was specifically designed and synthesized for the inhibition of the *E. coli* EntE A domain [98]. This compound showed stronger inhibition of EntE (*K*i = 4.5 nM) when compared to the inhibition of DhbE with DHB-AMS (*K*i = 85 nM). Furthermore, DHB-AMS exhibited higher activity with YbtE than with DhbE, suggesting that inhibitory susceptibility may vary depending on the specific enzyme [40,98].

**Figure 24:**
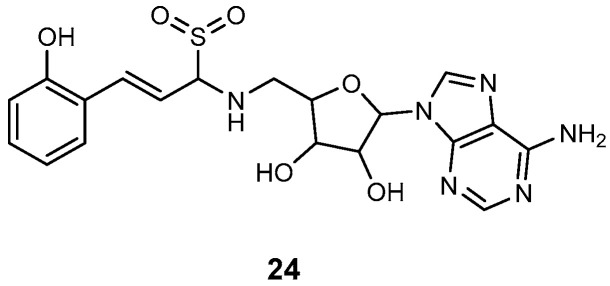
Vinylsulfonamide inhibitor **24**.

**Vinylsulfonamide inhibitors for capturing adenylation–carrier domain interactions.** The adenylation domains of NRPS enzymes undergo significant conformational changes during substrate activation and transfer. Initially, they activate an amino or hydroxy acid, followed by a large conformational shift to attach these acids to the carrier domain on the pantetheine group. Vinylsulfonamide inhibitors (**24,** Figure 24) are mechanism-based agents designed to covalently bind the carrier domain’s pantetheine group to a non-functional analogue of the AMS compounds. This covalent interaction effectively captures the adenylation–carrier domain interaction, interfering with the normal function of the NRPS system [34,99,100].

#### 4.1.3. An NRPS Enzyme Used as an Inhibitor

To explore whether microbial competition could lead to the discovery of new antifungal proteins, researchers screened cell-free extracts containing secreted or cytoplasmic proteins for antifungal properties against *Aspergillus fumigatus*. Among these extracts, a protein derived from *E. coli* exhibited significant inhibitory effects on fungal growth. Further analysis revealed that this protein was part of an NRPS within a siderophore biosynthetic cluster. Subsequent studies demonstrated that this protein inhibits siderophore production in *A. fumigatus*, suggesting a potential role in microbial competition and offering a novel avenue for antifungal development [101].

### 4.2. PKS Biosynthesis Inhibitors

Inhibiting PKS biosynthesis presents a significant challenge in drug development due to the structural and functional similarities between PKS and fatty acid synthase (FAS) enzymes. Both enzyme families are believed to have evolved from a common ancestor, leading to concerns that inhibitors targeting pathogenic PKS enzymes may also interfere with host FAS enzymes, complicating the development of selective drugs. As a result, PKS inhibitor research has shifted toward identifying and developing novel polyketides with therapeutic potential [102,103]. Polyketides constitute a diverse class of bioactive compounds, and natural product screening strategies have led to the commercialization of over 20 drugs, including the potent antimicrobials tetracycline and erythromycin. With advancements in microbial genome sequencing and the development of sophisticated genome mining tools, there has been a renewed effort to discover novel polyketides with pharmaceutical applications. These efforts are expected to expand the range of bioactive polyketides available for drug development [104].

**Figure 25:**
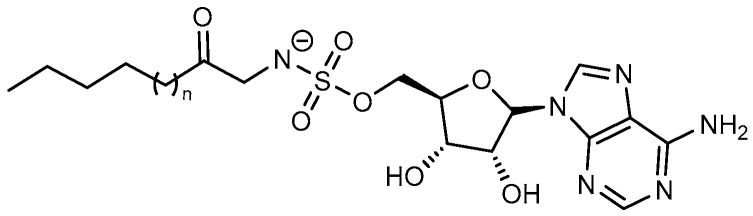
Fatty acyl-AMS inhibitors: hexanoyl-AMS (n = 1), lauryl-AMS (n = 7), arachidyl-AMS (n = 15).

Fatty acyl-AMP ligases (FAALs) have emerged as potential antibacterial targets due to their role in lipid virulence factor biosynthesis and metabolism in mycobacteria. FAALs facilitate the transfer of fatty acyl chains to an acyl carrier protein domain of a polyketide synthase, functioning similarly to NRPS adenylation domains. Conversely, fatty acyl-CoA ligases (FACLs) catalyse the transfer of acyl groups to CoA, resembling the activity of acyl-CoA synthetases. A study has identified fatty acyl-AMS analogues (Figure 25) as inhibitors of both FAALs and FACLs [105]. These analogues exhibited moderate antibacterial activity against *M. tuberculosis* and *M. smegmatis* in cell cultures [85,105].

### 4.3. Inhibitors Targeting NIS Biosynthesis

**Figure 26:**
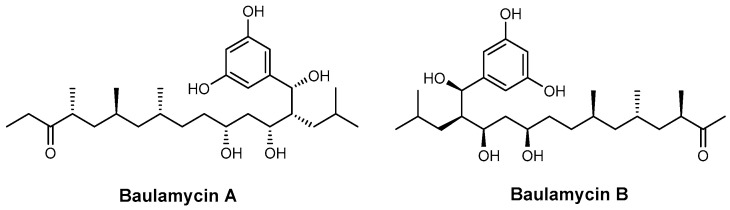
Structures of baulamycin A and baulamycin B.

Unlike many bacteria that use NRPS-dependent pathways, *S. aureus* synthesizes siderophores through an NRPS-independent pathway. This allows for the production of essential siderophores such as staphyloferrin B and petrobactin, which are crucial for survival under iron-limiting conditions. NIS synthetases, including SbnE in *S. aureus* and AsbA in *Bacillus anthracis*, catalyse the ATP-dependent attachment of citric acid to an amine alcohol or polyamine. This process involves citrate adenylation with pyrophosphate elimination, followed by linkage to the amine with AMP release. While this reaction is chemically similar to NRPS adenylation domains, it does not utilize a thiotemplate assembly line. To facilitate high-throughput screening for inhibitors, researchers developed an activity assay that converts pyrophosphate to inorganic phosphate. This screening led to the identification of baulamycins as competitive, reversible inhibitors of SbnE and AsbA. However, baulamycins inhibit pathogen growth in both iron-replete and iron-limiting conditions, suggesting a broader, non-specific mode of action [106,107]. Baulamycins A and B (Figure 26), marine microbial natural products, have been recognized as inhibitors of these NIS synthetases, with IC_50_ values ranging from approximately 5 to 200 μM [107]. Despite their inhibitory activity, their broad-spectrum effects raise concerns about specificity and therapeutic potential.

### 4.4. Inhibition of Siderophore Biosynthesis in Pathogenic Fungi

The opportunistic fungal pathogen *A. fumigatus* is a major cause of severe invasive fungal infections. Siderophore biosynthesis plays a vital role in its pathogenicity, facilitating iron acquisition under host-imposed iron restriction. The enzyme siderophore A (SidA) catalyses the first step in the biosynthesis of all four hydroxamate-containing siderophores in *A. fumigatus*. This reaction involves the hydroxylation of L-ornithine (L-Orn) to *N*^5^-L-hydroxyornithine, requiring oxygen and NADPH as cofactors [108,109].

**Figure 27:**
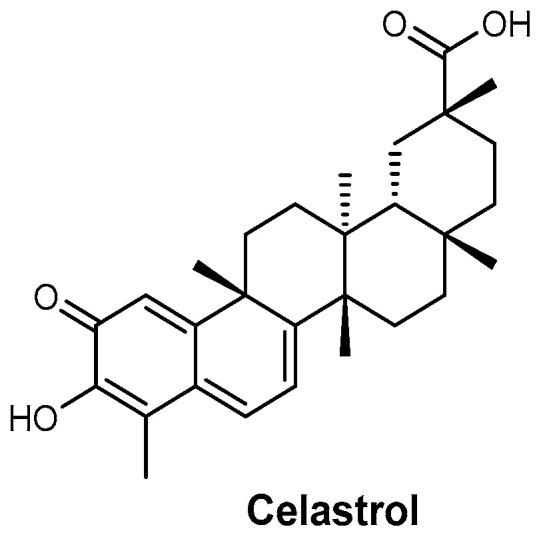
Structure of celastrol.

Celastrol (Figure 27), a naturally occurring quinone methide, has been identified as a noncompetitive inhibitor of SidA. By disrupting siderophore biosynthesis, celastrol effectively inhibits fungal growth, highlighting its potential as an antifungal agent [110,111].

Table 1 summarizes characterized inhibitors that interfere with siderophore biosynthesis, categorized by their specific enzymatic targets within the nonribosomal peptide synthetase (NRPS), polyketide synthase (PKS), and NIS (nonribosomal-independent siderophore) pathways. Information is provided on the inhibitory potency (IC_50_ values), the target species, and evidence from preclinical assays.

### 4.5. Nanoparticle Delivery Systems

Given the growing concerns about antibiotic resistance, alternative strategies targeting siderophore-mediated iron acquisition have garnered significant interest. The development of nanodrugs that selectively target multidrug-resistant bacteria while preserving beneficial microbiome components has emerged as a promising strategy to combat infections and mitigate antimicrobial resistance. Among these, nanoparticles (NPs) have emerged as promising agents due to their small size (<100 nm), high surface area-to-volume ratio, and unique antimicrobial properties. Metal nanoparticles, in particular, act as antibacterial agents by releasing metal ions that disrupt microbial metabolism and inhibit virulence factor expression. Moreover, nanoparticles offer advantages in targeted drug delivery and enhanced bioavailability [112,113]. *Pseudomonas aeruginosa* is a highly adaptable pathogen that utilizes siderophores, such as pyoverdine (PVD) and pyochelin (PCH), to acquire iron and enhance its virulence. Several studies have explored the potential of nanoparticles in disrupting *P. aeruginosa* virulence. For instance, iron nanoparticles derived from *Aloe vera* leaves exhibited the dose-dependent inhibition of siderophore production at concentrations exceeding 25 µM by penetrating the bacterial membrane and disrupting pyoverdine biosynthesis [114,115]. Chitosan-polypyrrole nanocomposites significantly suppressed PVD production by 91% at a concentration of 512 µg/mL. Likewise, phloroglucinol-zinc oxide nanoparticles displayed concentration-dependent inhibition, reaching a maximum reduction of 71.7% at 512 µg/mL. Fucoidan-gold nanoparticles, at 32 µg/mL, inhibited PVD formation in *P. aeruginosa* by approximately 95%. Furthermore, gold and zinc oxide nanoparticles demonstrated the dose-dependent suppression of PVD production, with inhibition rates of 55% and 85%, respectively, at 1024 µg/mL. In vitro cytotoxicity against the macrophage cell line RAW 264.7 was investigated. The nanoparticles showed no cytotoxicity at concentrations from 32 to 1024 μg/mL. However, cytotoxic effects were observed at 2048 μg/mL [115,116].

### 4.6. CRISPR-Based Approaches

CRISPR-based genome editing has emerged as a powerful tool for studying and manipulating bacterial genes, including those involved in siderophore biosynthesis. By precisely targeting and disrupting specific genes, researchers can investigate the roles of these genes in iron acquisition and pathogenicity, potentially leading to novel antimicrobial strategies. This strategy has been applied in *Bacillus mycoides* EC18, by interrupting two siderophore biosynthesis gene clusters via a CRISPR-Cas9 system [117]. This work demonstrated the influence of siderophore biosynthesis in bacterial interactions with plant hosts, highlighting the significant role of petrobactin in plant growth promotion and root colonization [117]. The engineered CRISPR-Cas9 system was used in another study where the authors were able to induce precise modifications in the genome of the *Streptomyces* species to enhance secondary metabolite production [118]. Although recent, this could be a promising approach to manipulate siderophore pathways for therapeutic purposes. The versatility of CRISPR-Cas9 in strain engineering is encouraging, and these examples illustrate its potential to dissect and modulate siderophore-related genes, paving the way for innovative antimicrobial therapies targeting iron acquisition mechanisms in pathogenic bacteria.

## 5. Challenges and Opportunities

While targeting siderophore biosynthesis has emerged as a promising antimicrobial strategy due to its critical role in microbial iron acquisition, it still faces several challenges. One of these challenges relies on the fact that microbes do not rely on just one way to obtain iron. Pathogens can produce several types of siderophores with specific iron binding groups (catecholates, hydroxamates, carboxylates, and mixed-type siderophores) and even possess alternative iron acquisition mechanisms, such as heme uptake or direct ferrous iron transport, which can compensate when a single pathway is inhibited. Additionally, the complex and modular nature of siderophore biosynthetic enzymes, like NRPS, PKS, and NIS, poses significant challenges for the design of specific and effective inhibitors. It is crucial to highlight that both the NIS biosynthetic pathway and the inhibition of siderophore biosynthesis in pathogenic fungi require further investigation as they remain largely unexplored. The NIS pathway, although well understood in bacteria, is less studied when compared to the NRPS and PKS pathways. Further research is needed to explore its unique aspects and potential applications, especially in fungi. Drug development and the identification of broad-spectrum agents are currently being somewhat restricted by the almost non-existent structural data available for many of these enzymes. Ultimately, microbial resistance will always be a challenge as pathogens may develop mutations, upregulate compensatory pathways or adapt metabolically in ways that render the efficacy of siderophore-targeting agents ineffective. Delivering inhibitors into bacterial cells, particularly within protective niches like biofilms or host tissues, primarily requires overcoming significant challenges related to stability, cellular uptake, and target specificity.

Despite these challenges, the inhibition of siderophore biosynthesis holds substantial opportunities. This strategy offers a promising therapeutic ally for fungal infections, but research is still in its early stages, as noticed due to the scarcity of literature examples. The complexity of these pathways and the diversity of siderophore structures across fungal species make the development of broad-spectrum inhibitors challenging. A deeper understanding of these processes is necessary to design more effective treatments, particularly as the emergence of drug-resistant infections continues to grow. Inhibiting siderophore production can also attenuate bacterial virulence without necessarily killing the pathogen, which may reduce selective pressure for resistance compared to traditional antibiotics. These inhibitors can be used in combination therapies, either to enhance the efficacy of existing antibiotics or to synergize with host immune responses by limiting iron availability. Another innovative application involves siderophore–antibiotic conjugates (“Trojan horse” strategies), where understanding and manipulating siderophore pathways can improve antibiotic delivery [12].

This strategy can be extended to other areas, as the inhibition of siderophore biosynthesis has potential for other applications beyond therapeutics. For example, its application in agriculture and biocontrol might be promising to obtain sustainable alternatives to chemical pesticides by targeting plant pathogens or in the modulation of iron competition in the rhizosphere. Additionally, emerging new strategies, such as the integration of CRISP-based tools, nanoparticle delivery systems, or even new approaches to synthetic biology, further expand the toolkit, enabling precise editing of siderophore genes for functional studies, resistance profiling, or the engineered production of novel antimicrobials.

## 6. Conclusions

Developing new antibiotics faces a challenge in achieving efficient drug delivery into bacteria cells, particularly for treating infections caused by Gram-negative pathogen. This difficulty stems from two primary factors: (i) the complex structure of the bacterial cell wall, which presents a diverse array of polarities, charges, and compositions that the drug must successfully navigate, and (ii) the challenge of integrating negatively charged functional groups into a single chemical entity, a key feature for effective recognition (reversible interactions) or bonding (irreversible/covalent) with bacterial cell wall components. To overcome these barriers, inhibitors must be designed with optimal physicochemical properties, including appropriate molecular size, charge distribution, lipophilicity, and membrane permeability. These characteristics are essential for ensuring efficient intracellular delivery and drug accumulation. Understanding siderophore biosynthesis is inherently complex, requiring the detailed elucidation of multiple enzymes, cofactors, and regulatory mechanisms involved in the pathway. A comprehensive understanding of these processes, along with siderophore uptake pathways and their interplay with other iron acquisition systems, is crucial for identifying viable drug targets and designing potent inhibitors. As in all antimicrobial strategies, the potential for resistance development remains a major concern when targeting siderophore biosynthesis. Bacteria and fungi may evolve resistance through mutations in target enzymes or proteins, upregulation of efflux pumps, or other adaptive mechanisms. To mitigate this risk, combination therapies or multi-target approaches aimed at inhibiting multiple components of the siderophore pathway may be necessary to preserve the long-term efficacy of future antimicrobials.

## Figures and Tables

**Table 1:** Inhibitors of siderophore biosynthesis.

Inhibitor	Enzyme	Enzyme Inhibition (IC_50_, μM)	Target Species	Preclinical Assays	Reference
Inhibitors targeting NRPS biosynthesis					
Phosphopantetheinyl transferase inhibitors					
**ML267**	Sfp AcpS	0.29 8.1	*B. subtilis*	In vitro inhibition In vitro ADME profile In vivo PK profile Mechanism of action	[67]
**Calmidazolium chloride**	VibB MAS	2.0 4.9	*V. cholerae* *M. tuberculosis*	In vitro inhibition Mechanism of action	[69]
**Sanguinarine chloride**	VibB MAS	22 4.9	*V. cholerae* *M. tuberculosis*	In vitro inhibition Mechanism of action	[69]
**1**	PptAb PcpS PptT	N.A. N.A. 2.3	*M. abscessus* *P. aeruginosa* *M. tuberculosis*	In vitro inhibition	[71] [71] [74]
**2**	PptT	2.5	*M. tuberculosis*	In vitro inhibition	[74]
**3**	PptT	3.8	*M. tuberculosis*	In vitro inhibition	[74]
**4**	PptT	0.71	*M. tuberculosis*	In vitro inhibition Cardiotoxicity studies	[73]
**5**	PptT	8.4	*M. tuberculosis*	In vitro inhibition	[73]
**6**	PptT	0.72	*M. tuberculosis*	In vitro inhibition Cardiotoxicity studies	[73]
**7**	PptT	0.065	*M. tuberculosis*	In vitro inhibition Cytotoxicity studies	[78]
**8**	PptT	0.018	*M. tuberculosis*	In vitro inhibition	[78]
**9**	PptT	8.29	*M. tuberculosis*	In vitro inhibition	[79]
**10**	PptT	0.49	*M. tuberculosis*	In vitro inhibition	[79]
**11**	PptT	173.16	*M. tuberculosis*	In vitro inhibition	[79]
**12**	PptT	9.28	*M. tuberculosis*	In vitro inhibition	[79]
**13**	PptT	12.29	*M. tuberculosis*	In vitro inhibition	[79]
**14**	PptT	28.58	*M. tuberculosis*	In vitro inhibition	[79]
Inhibitors of the adenylation domain					
**Salicyl-AMS**	MbtA YbtE PchD DhbE	10.7 14.7 12.5 N.A.	*M. tuberculosis* *Y. pestis* *P. aeruginosa* *B. subtilis*	In vitro inhibition In vivo efficacy and PK studies	[70] [70] [70] [82]
**15**	MbtA	N.A.	*M. tuberculosis*	In vitro inhibition	[83]
**L** **-Phenylalanyl-AMS**	GrsA SrfA-C	N.A.	*B. brevis* *B. subtilis*	In vitro inhibition Mechanism of action	[87]
**L** **-Leucyl-AMS**	GrsA SrfA-C	N.A.	*B. brevis* *B. subtilis*	In vitro inhibition Mechanism of action	[87]
**AA-AMS**	GrsA	N.A.	*A. miglanus*	In vitro inhibition and PK studies	[88]
**16**	GrsA	N.A.	*A. miglanus*	In vitro inhibition	[88]
**17**	HMWP2	N.A.	*Y. pestis*	In vitro inhibition	[91]
**18**	HMWP2	N.A.	*Y. pestis*	In vitro inhibition	[91]
**Alanyl-AMS**	HMWP2 (ATP–^32^PPi isotope exchange assay)	N.A.	*Y. pestis*	In vitro inhibition Mechanism of action	[91]
	HMWP2 (MesG assay)	1.15	*Y. pestis*	In vitro inhibition Mechanism of action	[90]
	IVT (ATP–^32^PPi isotope exchange assay)	0.16	*Y. pestis*	In vitro inhibition Mechanism of action	[91]
	IVT (MesG assay)	0.118	*Y. pestis*	In vitro inhibition Mechanism of action	[90]
**Lactyl-AMS**	HMWP2 (MesG assay) IVT (MesG assay)	>1000 166.4	*Y. pestis*	In vitro inhibition Mechanism of action	[90]
**Cysteyl-AMS**	HMWP2 (MesG assay) IVT (MesG assay)	0.04 3.27	*Y. pestis*	In vitro inhibition Mechanism of action	[90]
**α-Hydroxycysteyl-AMS**	HMWP2 (MesG assay) IVT (MesG assay)	16.53 221.5	*Y. pestis*	In vitro inhibition Mechanism of action	[90]
**Cyclo-alanylAMS**	HMWP2 (ATP–^32^PPi isotope exchange assay)	N.A.	*Y. pestis*	In vitro inhibition Mechanism of action	[91]
	HMWP2 (MesG assay)	0.34	*Y. pestis*	In vitro inhibition Mechanism of action	[90]
	IVT (ATP–^32^PPi isotope exchange assay)	>250	*Y. pestis*	In vitro inhibition Mechanism of action	[91]
	IVT (MesG assay)	>250	*Y. pestis*	In vitro inhibition Mechanism of action	[90]
**Cyclo-lactyl-AMS**	HMWP2 (MesG assay) IVT (MesG assay)	>1000 >250	*Y. pestis*	In vitro inhibition Mechanism of action	[90]
**19**	Mab-SaS	5.3	*M. abscessus*	In vitro inhibition Cytotoxicity studies	[29]
**20**	Mab-SaS	2.6	*M. abscessus*	In vitro inhibition Cytotoxicity studies	[94]
**21**	MbtI	6.3	*M. tuberculosis*	In vitro inhibition	[95]
**22**	MbtI	12	*M. tuberculosis*	In vitro inhibition	[96]
**DHB-AMS**	DhbE YbtE	N.A.	*B. subtilis* *Y. pestis*	In vitro inhibition and PK studies	[82]
**23**	DhbE YbtE	N.A.	*B. subtilis* *Y. pestis*	In vitro inhibition	[98]
Inhibitors targeting PKS biosynthesis					
**Hexanoyl-AMS**	FAAL28 FACL19	N.A.	*M. tuberculosis*	In vitro inhibition Mechanism of action	[105]
**Lauryl-AMS**	FAAL28 FACL19		*M. tuberculosis*	In vitro inhibition Mechanism of action	[105]
**Arachidyl-AMS**	FAAL28 FACL19		*M. tuberculosis*	In vitro inhibition Mechanism of action	[105]
Inhibitors targeting NIS biosynthesis					
**Baulamycin A**	SbnE AsbA AsbB	4.8 μM 180 μM >1000	*S. aureus* *B. anthracis* *B. anthracis*	In vitro inhibition Mechanism of action	[107]
**Baulamycin B**	SbnE AsbA AsbB	19 μM 200 μM >1000 μM	*S. aureus* *B. anthracis* *B. anthracis*	In vitro inhibition Mechanism of action	[107]
Inhibition of siderophore biosynthesis in pathogenic fungi					
**Celastrol**	SidA	11 μM	*A. fumigatus*	In vitro inhibition Mechanism of action	[110]

Note: ADME: absorption, distribution, metabolism, and excretion; PK: pharmacokinetics; IC_50_: half maximal inhibitory concentration; N.A.—not available.

## Data Availability

No new data were created or analysed in this study. Data sharing is not applicable to this article.

## Abbreviations

The following abbreviations are used in this manuscript:

A	Adenylation
ABC	ATP-binding cassette
ACP	Acyl carrier proteins
AMP	Adenosine monophosphate
AMS	Adenosine monosulfamate
AT	Acyltransferase domain
ATP	Adenosine triphosphate
BCG	Bacillus Calmette-Guerin
C	Condensation
CM	Cytosolic membrane
CoA	Coenzime A
Cy	Cyclization
DHB	Dihydroxybenzoate
DNA	Deoxyribonucleic Acid
E	Epimerization
EC_50_	Half maximal effective concentration
EMA	European Medicines Agency
FAAL	Fatty acyl-AMP ligase
FACL	Fatty acyl-CoA ligase
FAS	Fatty acid synthase
FDA	Food and Drug Administration
Fur	Ferric uptake regulator
IC_50_	Half maximal inhibitory concentration
K	Ketosynthase domain
Ki	Inhibitor constant
MbtA	Mycobacterium tuberculosis salicylate adenylation enzyme
MbtI	Mycobacterium tuberculosis salicylate synthase
MFP	Membrane fusion protein
MFS	Major facilitator superfamily
Mt	Methylation
NADH	1,4-Dihydronicotinamide adenine dinucleotide
NADPH	Nicotinamide adenine dinucleotide phosphate
NIS	NRPS-independent
NRPS	Nonribosomal peptide synthetase
NTM	Non-tuberculous mycobacteria
NTP	Nucleoside triphosphate
OM	Outer membrane
OMF	Outer membrane factor
Ox	Oxidation
P	Carrier domain
PBP	Periplasmic binding proteins
PCP	Peptidyl carrier protein
PKS	Polyketide synthase
PPTase	Phosphopantetheinyl transferases
RNA	Ribonucleic acid
RND	Gram-negative-specific resistance/nodulation/cell division
SAL-AMS	5′-O-[N-(salicyl)-sulfamoyl] adenosine
SBP	Siderophore-binding protein
T	Thiolation
TBDT	TonB-dependent transporter
TE	Thioesterase
tRNA	Transfer ribonucleic acid
